# Health Tract, No. 225

**Published:** 1865

**Authors:** 


### "DIRTY CHILDREN."

There is an undefined impression left on the minds of many, in passing a group of chubby-looking children playing in the street or by the roadside, bare-footed, bare-headed, and ragged, begrimed with dust or mud, that "dirt must be healthy." And when there is noticed around the cabins of the country poor, or the shanties in the city outskirts, a crowd of ragamuffin urchins of all sizes, like the regular gradations of a ladder, another notion is almost formed, in distinct words, that "poverty is healthy" as well as dirt, as the having a house full of children is taken as proof of vigorous constitutions on the part of the toiling parents. Taking New-York City as a guide, the official reports for 1863 show that of every ten deaths, seven are foreign, although just half the population is foreign-born; and as a class, foreigners are the poorest and the filthiest of all American large seaboard cities; of course, there are notable exceptions. It is known that those who live on their daily wages, average eleven years less of life than those who are well to do. So that poverty is as far from being healthful, as it is from being agreeable. Of one thousand children dying under one year old, nearly three fourths were born of foreign parents; two thirds of all the children dying on the day of their birth, were of foreign parentage. Of those dying from one to five years old, three fourths were born of poor people. Of nine children, Queen Victoria lost none. The constitutions of royal pairs may not be as vigorous as those of two young laborers, but exemption from exhausting toil, and their ability to command roomy residences, well-ventilated chambers, and the strictest personal cleanliness from earliest infancy, more than counterbalance other unfavoring circumstances. So far then from poverty and filth being elements of health and long life, they are the very reverse; they directly induce premature death as to grown-up persons, and sow the seeds of fatal diseases in innocent childhood. During the first week of August, 1864, in New-York City, four hundred and forty-four children died; of which four hundred and four were of foreign parentage, and only forty were born of native parents; that is, ninety per cent of the children dying in New-York, nine out of ten, are from the abodes of poverty and untidiness. Fifteen thousand children died in New-York during 1864, of which eighty-eight per cent were the children of foreigners, and twelve per cent of native parents.

